# No SARS-CoV-2 detection in the German CAPNETZ cohort of community acquired pneumonia before COVID-19 peak in March 2020

**DOI:** 10.1007/s15010-020-01471-y

**Published:** 2020-07-03

**Authors:** Marcus Panning, Julius Wiener, Kathrin Rothe, Jochen Schneider, Mathias W. Pletz, Gernot Rohde, Jan Rupp, Martin Witzenrath, Christoph D. Spinner, Members of the CAPNETZ study group, M. Dreher, C. Cornelissen, W. Knüppel, D. Stolz, N. Suttorp, W. Bauer, A. Mikolajewska, M. Witzenrath, W. Pankow, S. Gläser, D. Thiemig, M. Prediger, S. Schmager, M. Kolditz, B. Schulte-Hubbert, S. Langner, G. Rohde, C. Bellinghausen, M. Panning, C. Hoffmann, T. Welte, J. Freise, G. Barten, W. Kröner, M. Nawrocki, J. Naim, T. Illig, N. Klopp, M. Pletz, C. Kroegel, B. Schleenvoigt, C. Forstner, A. Moeser, D. Drömann, P. Parschke, K. Franzen, J. Rupp, N. Käding, M. Wouters, K. Walraven, D. Braeken, C. Spinner, A. Zaruchas, Schaberg, D. Heigener, I. Hering, W. Albrich, F. Waldeck, F. Rassouli, S. Baldesberger, S. Stenger, M. Wallner, H. Burgmann, L. Traby

**Affiliations:** 1 Institute of Virology, Medical Center - University of Freiburg, Faculty of Medicine, University of Freiburg, Hermann-Herder-Str. 11, 79014 Freiburg, Germany; 2 Technical University Munich, School of Medicine, Institute for Medical Microbiology, Immunology and Hygiene, Munich, Germany; 3 Department of Internal Medicine II, School of Medicine, Technical University Munich, University Hospital rechts der Isar, Munich, Germany; 4 Institute of Infectious Diseases and Infection Control, Jena University Hospital/Friedrich-Schiller-University Jena, Jena, Germany; 5 Department of Respiratory Medicine, Medical Clinic I, University Hospital, Goethe University Frankfurt, Frankfurt, Germany; 6 Department of Infectious Diseases and Microbiology, University Hospital Schleswig–Holstein, Lübeck, Germany; 7 Department of Infectious Diseases and Pulmonary Medicine and Division of Pulmonary Inflammation, Charité - Universitätsmedizin Berlin, Freie Universität Berlin, Humboldt-Universität zu Berlin, and Berlin Institute of Health, Berlin, Germany; 8 CAPNETZ STIFTUNG, Hannover, Germany; 9 Biomedical Research in Endstage and Obstructive Lung Disease Hannover (BREATH), Member of the German Center for Lung Research (DZL), Hannover, Germany; 10 German Center for Lung Research (DZL), Hannover, Germany

**Keywords:** SARS-CoV-2, Community acquired pneumonia, Respiratory pathogens, Epidemiology, Multiplex RT-PCR

## Abstract

**Purpose:**

The first SARS-CoV-2 cases in Europe were reported in January 2020. Recently, concern arose on unrecognized infections before this date. For a better understanding of the pandemic, we retrospectively analyzed patient samples for SARS-CoV-2 from the prospective CAPNETZ study cohort.

**Methods:**

We used nasopharyngeal swab samples from a cohort of well characterized patients with community acquired pneumonia of the CAPNETZ study group, recruited from different geographic regions across Germany, Austria, the Netherlands, and Switzerland between 02nd December 2019 and 28th April 2020. Multiplex real-time RT-PCR for a broad range of respiratory pathogens and SARS-CoV-2 real-time RT-PCR were performed on all samples.

**Results:**

In our cohort, respiratory pathogens other than SARS-CoV-2 were detected in 21.5% (42/195) of patients with rhinovirus as the most frequently detected pathogen. The detection rate increased to 29.7% (58/195) when SARS-CoV-2 was included. No SARS-CoV-2 positive sample was detected before end of March 2020.

**Conclusions:**

Respiratory viral pathogens accounted for a considerable number of positive results but no SARS-CoV-2 case was identified before the end of March 2020.

## Introduction

The novel coronavirus, severe acute respiratory syndrome coronavirus-2 (SARS-CoV-2), emerged in China in late 2019 [1]. Recent genomic data suggests that it is of zoonotic origin and crossed the species barrier from an as-of-yet unknown animal host to humans in late November 2019 to early December 2019 [2]. Date of onset of disease of the first described SARS-CoV-2 patient in Wuhan, China was 1 December 2019 [3]. In Europe, the first cases were detected in France on 17 January 2020 and shortly after in Germany and elsewhere [4]. However, modelling studies suggest that individuals exposed to SARS-CoV-2 might have been traveling internationally from mainland China before travel restrictions were in place. [5]. This gives rise to the notion of unexpected introductions within China and beyond which remained unrecognized. Here, we used prospectively collected samples from well characterized patients with community acquired pneumonia to test the hypothesis of cryptic spread of SARS-CoV-2 before the end of January in Germany.

## Methods

We included 195 nasopharyngeal swab samples which were submitted to the Institute of Virology, Freiburg, Germany between 2 December 2019 (calendar week 48) and 28 April 2020 (calendar week 17) in this analysis. Samples were retrieved in the framework of CAPENTZ [competence network for community-acquired pneumonia (CAP)] study group. In brief, inclusion criteria were age ≥ 18 years and community-acquired pneumonia was confirmed by radiological proof of a new lung infiltrate plus ≥ one of the following: cough, purulent (off-white, yellow or green and opaque) sputum, fever (≥ 38.3 °C), and auscultatory findings consistent with pneumonia. Each sample resembles one individual patient. Patients were recruited in local study centers in Austria, Germany, the Netherlands, and Switzerland: Bad Arolsen (*n* = 13), Berlin (Charité Campus Benjamin Franklin *n* = 15; Charité Virchow Klinikum *n* = 4; Vivantes Klinikum Neukölln *n* = 19) Cologne (*n* = 1), Cottbus (*n* = 20), Dortmund (*n* = 2), Dresden (*n* = 4), Frankfurt (*n* = 11), Gerlingen (*n* = 3), Hamburg (*n* = 2), Hannover (*n* = 4), Lübeck (*n* = 19), Munich (*n* = 26), Paderborn (*n* = 5), Rotenburg (*n* = 13), Basle (*n* = 14; Switzerland), St Gallen (*n* = 1; Switzerland, Maastricht (*n* = 3; the Netherlands), and Vienna (*n* = 11; Austria). All samples were immediately analyzed upon receipt using a multiplex real-time RT-PCR panel according to Bierbaum et al. [6]. This included testing for respiratory viruses [adenovirus, bocavirus, coronavirus (CoV) OC43, CoV 229E, CoV HKU1, CoV NL63, enterovirus, influenza virus A + B, human metapneumonvirus, parainfluenza virus 1–4, human parechovirus, respiratory syncytial virus A + B, rhinovirus] and atypical bacteria (*Bordetella pertussis, Legionella pneumophila, Mycoplasma pneumoniae*). Testing for SARS-CoV-2 was performed retrospectively using the RealStar SARS-CoV-2 RT-PCR (Altona Diagnostics, Hamburg, Germany) according to the manufacturer’s recommendation.

## Results

**Fig. 1:**
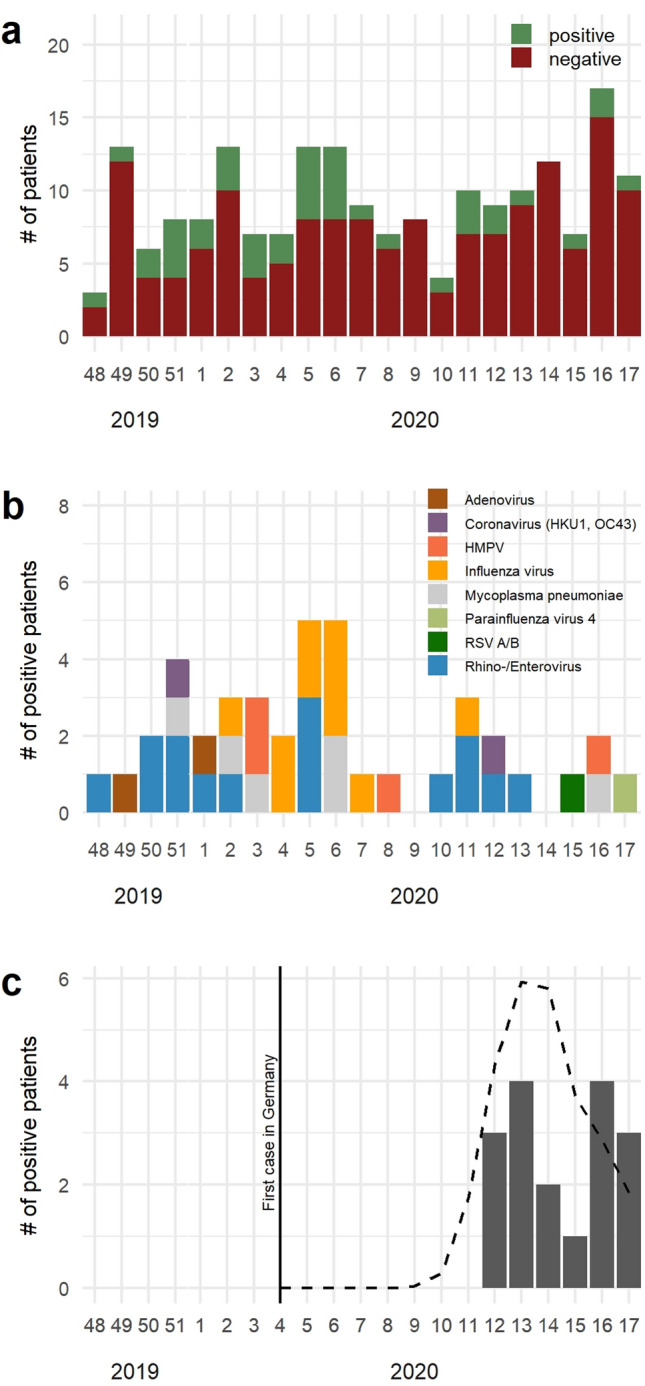
**a** Total number of patients and fraction of patients tested positive or negative for a respiratory pathogen using multiplex real-time RT-PCR (excluding SARS-CoV-2) per calendar week from week 48/2019 to 17/2020. **b** Number of patients tested positive for a respiratory pathogen per calendar week. **c** SARS-CoV-2 positive patients from calendar week 48/2019 to 17/2020. Broken line indicates epidemic curve of reported cases in Germany, solid vertical line denotes first reported case in Germany

A total of 195 samples were included with 3 to 18 patients recruited per calendar week (Fig. 1a). Median age of patients was 65.5 years (range 21–98 years), and 44 (29%) were females (missing data from 41 patients). Of these, 42 (21.5%) patients tested positive for a respiratory pathogen included in the multiplex PCR panel. Rhinovirus (14/195; 7.2%) was the most frequently detected virus, followed by influenza A virus (10/195; 5%), and HMPV (4/195; 2%). Only two CoV were identified: one patient with CoV HKU1 and one with CoV OC43. Other viruses detected were adenovirus (*n *= 2), enterovirus (*n *= 1), HMPV (*n *= 4), parainfluenza virus 4 (*n *= 1), and RSV A/B (*n *= 1), respectively. Of the atypical bacteria included in the panel, only *Mycoplasma pneumoniae* was detected in 6/195 (3%) patients. No co-detections (viral–viral or viral-bacterial) were found. Detection rates of positive samples peaked in calendar week 6 and rapidly declined hereafter (Fig. 1b).

Testing for SARS-CoV-2 RNA revealed another 17 positive results bringing the total number of positive results to 58/195 (29.7%) (Fig. 1c). The first SARS-CoV-2 positive sample in this cohort was detected on 24 March 2020 and originated from a patient already tested positive in the local study center due to a high clinical suspicion of coronavirus disease-19 (COVID-19). All SARS-CoV-2 positive patients were recruited in German local study centers. No single patient tested SARS-CoV-2 RNA positive before this date in our cohort. No co-detections were found in SARS-CoV-2 positive samples at baseline of sampling.

## Discussion

A better understanding of the epidemiology of SARS-CoV-2 is mandatory for Public Health preparedness and response. No evidence of SARS-CoV-2 before the end of March 2020 in samples of patients with suspected community acquired pneumonia was seen in our cohort, corresponding to the clinically observed COVID-19 peak in Germany. The first COVID-19 pandemic peak was also observed in our rather small cohort of CAP patients. We used a cohort of well characterized patients, which met the current clinical case definition for COVID-19, i.e. pneumonia or acute respiratory tract infection of any severity. However, we did not include a review of medical records and we were not able to clearly differentiate between typical or atypical pneumonia based on radiological criteria due to a lack of available data. The multicenter design of our study adds further significance to our findings. Our approach supports the importance of well characterized patient cohorts and shows the value of biobanks. In light of a massive increase in testing capacity it remains speculative if cryptic COVID-19 has gone undetected as many laboratories in Germany have rapidly adopted SARS-CoV-2 testing protocols. In fact, our study supports the hypothesis, that no notable numbers of undetected SARS-CoV-2 infections were present before the major outbreak in Germany. A subset of patients (15%) was recruited in neighboring countries, i.e. Austria, the Netherlands, and Switzerland. No SARS-CoV-2 positive patient was detected among these patients, but warrants further studies with larger sample sizes.

Notably, rhinovirus was the most frequently detected non-SARS-CoV-2 respiratory virus in our study. This matches well with data from the large EPIC study [7]. However, the exact role of rhinovirus in upper respiratory tract samples of patients with CAP is still a matter of debate. Influenza virus was the second most prevalent viral pathogen, which coincides with the peak of the influenza season in the northern hemisphere around calendar week 6/2020. Of note, the 2019/20 influenza season was only moderate and had a shorter duration compared with previous seasons. The reasons for this are manifold and include prevailing influenza virus strains, background immunity in the population, vaccine uptake and efficacy but it seems likely that the rapid decline at the end of the season was also influenced by the COVID-19 Public Health measures. This abrupt decline was also observed in surveillance and reporting systems for acute respiratory tract infections (grippeweb.rki.de) of the German National Public Health Robert-Koch-Institute [8].

As a limitation, we did not recruit and analyze asymptomatic controls by protocol definition of the CAPNETZ study. This was a rather small scale study and analyzing larger cohorts including children in further studies is warranted. The role of co-detections of respiratory pathogens in COVID-19 patients needs to be addressed in more detail.

## Conclusion

We identified respiratory pathogens in 21.5% of patients with community acquired pneumonia in the national CAPNETZ cohort using molecular testing only. SARS-CoV-2 was not detected retrospectively in samples before COVID-19 peaked in Germany giving rise to the notion that SARS-CoV-2 did not initiate unrecognized transmission chains before the end of January.
